# Preclinical Pharmacokinetic Study and Lung Penetration of a Coumarin Extracted from *Zanthoxylum tingoassuiba*

**DOI:** 10.3390/pharmaceutics16060714

**Published:** 2024-05-26

**Authors:** Valdeene Vieira Santos, Matheus Antônio da Hora Borges, Karoline Cristina Jatobá da Silva, Rafael dos Santos Costa, Renan Fernandes do Espírito Santo, Eudes da Silva Velozo, Cristiane Flora Villarreal, Francine Johansson Azeredo

**Affiliations:** 1 Pharmacy Graduate Program, College of Pharmacy, Federal University of Bahia, Salvador 40170-115, Brazil; enevieira@hotmail.com (V.V.S.); mborges976@gmail.com (M.A.d.H.B.);; 2 College of Pharmacy, Federal University of Bahia, Salvador 40170-115, Brazil; 3 Center for Research on Aging, Department of Neural and Pain Sciences, University of Maryland, Baltimore, MD 21201, USA; 4 Center for Pharmacometrics and Systems Pharmacology, Department of Pharmaceutics, College of Pharmacy, University of Florida, Orlando, FL 32827, USA

**Keywords:** bioanalytical method validation, natural products, noncompartmental analysis

## Abstract

The compound 6-methoxyseselin, derived from *Zanthoxylum tingoassuiba*, demonstrates various therapeutic properties, including vasorelaxation, antinociceptive, anti-inflammatory, and immunomodulatory effects, along with recently discovered antiasthmatic properties. This study aimed to evaluate its preclinical pharmacokinetics and pulmonary delivery in Balb/c mice. The method involved administering the compound via inhalation and intravenous routes, followed by blood sample collection for analysis using high-performance liquid chromatography with diode array detection (HPLC-DAD). The results indicated good linearity, precision, accuracy, and stability of the compound in the biological samples. Pharmacokinetic parameters such as the rate of elimination, half-life, clearance, volume of distribution, area under the curve, and mean residence time were determined for both administration routes, showing similar profiles. The lung concentrations were notably higher than the plasma concentrations, indicating significant lung penetration. These findings suggest 6-methoxyseselin as a promising candidate for new anti-asthmatic drugs, supported by its favorable pharmacokinetic profiles and high lung penetration factors. This study represents the first exploration of the pharmacokinetics and pulmonary delivery of 6-methoxyseselin in mice, highlighting its potential for further drug development.

## 1. Introduction

Natural products were and still are a great source of new drugs. Because of the wide structural variety, it allows different substances found in different living beings to become targets of study for the production process and development of new drugs [1,2]. *Zanthoxylum*, a genus of plants belonging to the *Rutaceae* family, is widely used traditionally as a medical plant due to the various therapeutic effects reported [3]. This extensive range of effects promoted by this plant is possible due to the diverse number of phytochemical groups present throughout the plant [4]. In studies, several species of the *Zanthoxylum* genus have shown a variety of coumarins present in their roots, which have reported pharmacological effects [3,4,5].

The *Zanthoxylum tingoassuiba* species, native to Brazil, has several effects associated with the coumarins present in its roots [6,7,8,9]. Coumarins, a class of compounds, possess significant pharmacological properties. Among natural compounds, they are considered crucial [10,11]. Some coumarins have approved clinical applications, such as being used as anticoagulants, anti-tumor agents, and anti-inflammatory substances [10,12,13].

**Figure 1:**
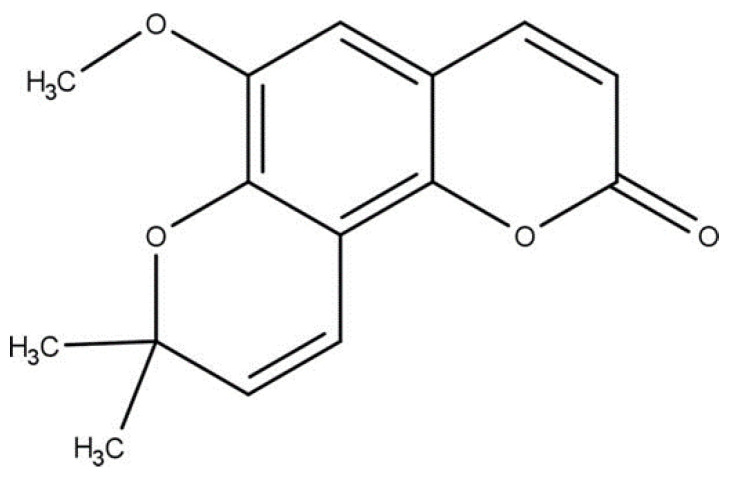
Chemical structure of 6-methoxyseselin.

6-Methoxyseselin (Figure 1) is one of these coumarins that can be isolated and shows promising pharmacological activities. Studies show that this compound induces potent vasorelaxation in arteries isolated from rats [8,9], in addition to antinociceptive, anti-inflammatory, and immunomodulatory properties [14]. Recently, the antiasthmatic properties have been demonstrated. Inhaled 6-methoxyseselin reduced, in a dose-dependent manner, key pathophysiological events of experimental asthma, with similar efficacy to systemic dexamethasone, considered the standard gold drug [15]. These data highlight the potential of coumarin as a candidate for the drug discovery process of new anti-asthmatic drugs.

Given these effects, 6-methoxyseselin presents itself as a promising alternative to the currently utilized drugs. As a result, it is necessary to conduct preclinical pharmacokinetic investigations aimed at characterizing 6-methoxyseselin’s concentration–time profile, determining its pharmacokinetic parameters, and formulating an appropriate dosage regimen. However, the execution of pharmacokinetic studies hinges upon the preliminary development and validation of a quantification method for 6-methoxyseselin in biological samples.

Consequently, this study aimed to evaluate 6-methoxyseselin’s preclinical pharmacokinetics (PK) and pulmonary delivery in Balb/c mice. To achieve this aim, we needed to devise and validate a straightforward yet highly sensitive technique for quantifying 6-methoxyseselin in plasma samples. This was achieved using high-performance liquid chromatography (HPLC) coupled with photodiode array detector (DAD) technology. This method holds the potential for future utilization in clinical pharmacokinetic investigations. After its development, the technique was employed in a preliminary pharmacokinetic inquiry after the intravenous and inhalation administration of 6-methoxyseselin in mice, where its efficacy was demonstrated.

## 2. Materials and Methods

### 2.1. Chemicals and Reagents

The current research is a continuation of previously published work [6,14,15]. Briefly, 6-methoxyseselin was identified and isolated from the roots of *Zanthoxylum tingoassuiba* A. St. Hil (Rutaceae) by the Medical Research Laboratory (LAPEMM) of the Federal University of Bahia (UFBA) (Brazil), which were collected in Feira de Santana, Brazil, 12°12′52.9″ S, 38°52′44.1″ W. A voucher specimen (no. 88005) was identified and deposited at the Alexandre Leal Costa Herbarium (ALCB) at UFBA. HPLC-grade methanol was purchased from J. T. Baker (Phillipsburg, NJ, USA). Ultrapure water was obtained from Thermo Scientific Barnstead MicroPure (Waltham, MA, USA). 6-Methoxyseselin was extracted, isolated, and identified as described by Espírito-Santo (2017) [14]. Briefly, in the extraction process, root bark (RB) and root heartwood (RH) obtained from *Z. tingoassuiba* were separated, ground, and extracted with hexane and methanol. The methanol extract underwent fractionation using a CHCl3:MeOH:5%HCl (5:5:3) partition. The organic layer was dried under reduced pressure, yielding the final fraction, which was further recrystallized with methanol, resulting in a yellow powder. This powder was subjected to NMR ^1^H and ^13^C, and the *m*/*z* value was determined through HPLC/MS [12]. After a thorough analysis and comparison with the literature data, 6-methoxyseselin was identified, as previously described [6].

### 2.2. Chromatography System and Chromatographic Conditions

The HPLC system consisted of a Shimadzu^®^ chromatograph, consisting of LC-6AD pumps, SPD-M20A detector PAD, CBM-20A controller, DGU-20A degasser, and LC Solution software version; 7.1, Japan. The separation took place using a C18 Kromasil^®^ column (250 mm; 4.6 mm; 5 µm 100 Å), as well as elution with an isocratic flow of 1.0 mL/min using a mixture of water (45%) and methanol (55%). The injection volume was 20 µL. All of the chromatograms were monitored using absorption lengths of 228 nm, which showed the best detection of the compound when a UV scanning spectrum was performed.

### 2.3. Calibration Curves and Quality Control Samples

The stock solution was prepared using 6.0 mg of 6-methoxyseselin dissolved in 10.0 mL of methanol and subjected to an ultrasound bath for 5 min. Then, the working solutions were designed for the calibration curve diluted in methanol from the stock solution, reaching the following concentrations: 50, 25, 12.5, 6.25, 3.125, and 1.5625 µg/mL. Calibration curves were established using 90 µL of mice plasma with 10 µL of the solutions to yield concentrations of 5, 2.5, 1.25, 0.625, 0.3125, 0.15625 µg/mL. A standard solution was used to prepare the quality control (QC) solutions, which were solubilized in methanol at concentrations of 40, 10, 5, and 1.5625 µg/mL. From the solutions designed above, 10.0 µL aliquots were used and diluted in 90.0 µL of plasma. In this way, final solutions were obtained at concentrations of 4.0, 1.0, 0.5, and 0.15625 µg/mL for the high-quality control (HQC), medium quality control (MQC), low-quality control (LQC), and lower limit of quantitation (LLOQ) respectively.

**Figure 2:**
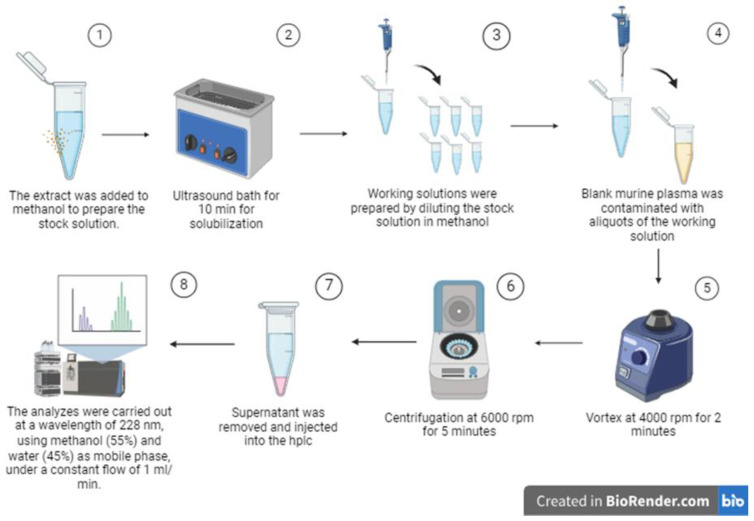
Quality control and calibration curve sample preparation.

The extraction of biological matrices was performed by adding 200 µL of methanol, homogenized by vertexing at 4000 rpm for 2 min, and, after shaking, it was centrifuged at 6000 rpm for 5 min. The samples were filtered through 3 mm PVDF filters, with an average porosity of 0.45 µm. The process is shown in Figure 2.

### 2.4. Methods Validation

The validation of the method followed the Bioanalytical Method Validation guide of the Food and Drug Administration (FDA), analyzing the parameters of selectivity, accuracy, precision, linearity, recovery, and stability [16].

#### 2.4.1. Selectivity

The method’s selectivity was demonstrated by analyzing biological samples of blank plasma compared to the chromatograms of mice plasma spiked with 6-methoxyseselin to prove that the plasma components did not interfere with the quantification of the analyte. Plasma samples from individual sources were evaluated at least six times [16].

#### 2.4.2. Sensitivity

Sensitivity was defined through LLOQ analysis, with an accuracy of ±20% of the nominal concentration and precision, with a coefficient of variation (CV) limit between ±20% for the intra and inter-day runs [16].

#### 2.4.3. Accuracy and Precision

The accuracy and precision were assessed across the entire quantification range, specifically on LLOQ, low, medium, and high QCs. Six replicates of LLOQ and QC samples were analyzed on two consecutive days (3 replicates per day) to determine the intra-day and inter-day, expressed as CV and relative standard error (RSE). Acceptance criteria were intra-day and inter-day accuracy within ±15% RSE of the nominal concentrations, except for LLOQ, which may be ±20%, and intra-day and inter-day accuracy not exceeding ±15% CV, except for the LLOQ, which was allowed to be within ±20% CV [16].

#### 2.4.4. Linearity

Linearity assessment was conducted via calibration curves, wherein the peak area was juxtaposed against its nominal concentration. This analysis ensued after selecting an appropriate quantification range and calibration standard concentrations aligned with anticipated study levels. Inclusive of a blank sample, the calibration standards spanned six distinct concentration levels, encompassing the lower limit of quantification (LLOQ). A linear regression model was employed to delineate the concentration–response relationship. This yielded a correlation coefficient (R^2^) surpassing 0.99. Moreover, the deviation of calibrators, excluding zero, from their respective nominal concentrations was confined within ±15%, barring LLOQ, where a tolerance of ±20% was permissible across each validation iteration [16].

#### 2.4.5. Recovery

Recovery (%) in the plasma samples was determined by analyzing the peak areas from the samples extracted with low, medium, and high QC concentrations versus the chromatographic peak areas from samples with 6-methoxyseselin dissolved directly in the solvent [16].

#### 2.4.6. Stability

Stability assays were performed in triplicate with the QC samples (LQC and HQC) by adding 6-methoxyseselin to mice plasma. The stability after 30, 45, and 60 days was performed with samples stored at −18 °C. 6-methoxyseselin was considered stable in plasma for the period evaluated when the accuracy was within acceptable limits (RSE within ±15%) [16].

### 2.5. Pharmacokinetics Study in Mice

The pharmacokinetic analysis was carried out with Balb/c mice (~30 g) obtained from the Animal Facilities at Oswaldo Cruz Foundation (Fiocruz, Bahia, Brazil) and kept in a room with environmental control (22 ± 2 °C and 12:12 h light–dark cycle), and with free access to water and food until the day of the experiment. Animal care and handling procedures were in strict accordance with the recommendations in the Guide for the Care and Use of Laboratory Animals of the National Institute of Health and the Brazilian College of Animal Experimentation. The protocol was approved by the Institutional Animal Care and Use Committee of FIOCRUZ (CEUA/FIOCRUZ, permit number: L-IGM-013/2021).

The compound was administered to the mice (n = 3 per time point) by inhalation and intravenous routes at 50 and 15 mg/kg, respectively. The intravenous administration was conducted through the lateral tail vein, and the inhalation dosing was conducted using inhalation exposure chambers. The nebulizer device was operated according to the manufacturer’s specifications [15]. The blood samples were collected 0.083, 0.25, 0.5, 0.75, 1, 2, and 4 h after both administrations. The blood samples were centrifuged for plasma separation (6000 rpm, 10 min, 8 °C) and stored at −18 °C until analysis.

The non-compartmental pharmacokinetic parameters of both studies were estimated using PKanalix^®^ Suite 2020R1 software (Lixoft^®^, Antony, France). The determined PK parameters included the elimination rate constant (ke), the elimination half-life (t1/2), the area under the concentration–time curve (AUC) calculated by the linear trapezoidal rule, the mean residence time (MRT), the total clearance (CL), and the volume of distribution (Vd) [17].

### 2.6. Lung Distribution in Mice

The lung distribution experiment used Male Balb/c (~30 g). After intravenous bolus and inhalation administration of 15 mg/kg and 50 mg/kg, respectively, of 6-methoxyseselin, three animals were sacrificed at 0.5 h, 1 h, 2 h, and 4 h, each. The lung samples were removed, gently blotted dry, weighed, and frozen at −80 °C until processing [18]. On the day of the analysis, tissue samples were allowed to thaw and were weighed, and 2 mL of methanol was added per gram of tissue. The tissues were homogenized for approximately 5 min, and the homogenates were then transferred to Eppendorf^®^ tubes and centrifuged at 3500× *g* for 15 min at 4 °C. A volume of supernatant was then processed similarly to the plasma samples and analyzed by HPLC-DAD. The lung penetration was calculated using the AUC lung/AUC plasma ratio. The trapezoidal method was used to estimate both AUCs.

## 3. Results

### 3.1. Selectivity

**Figure 3:**
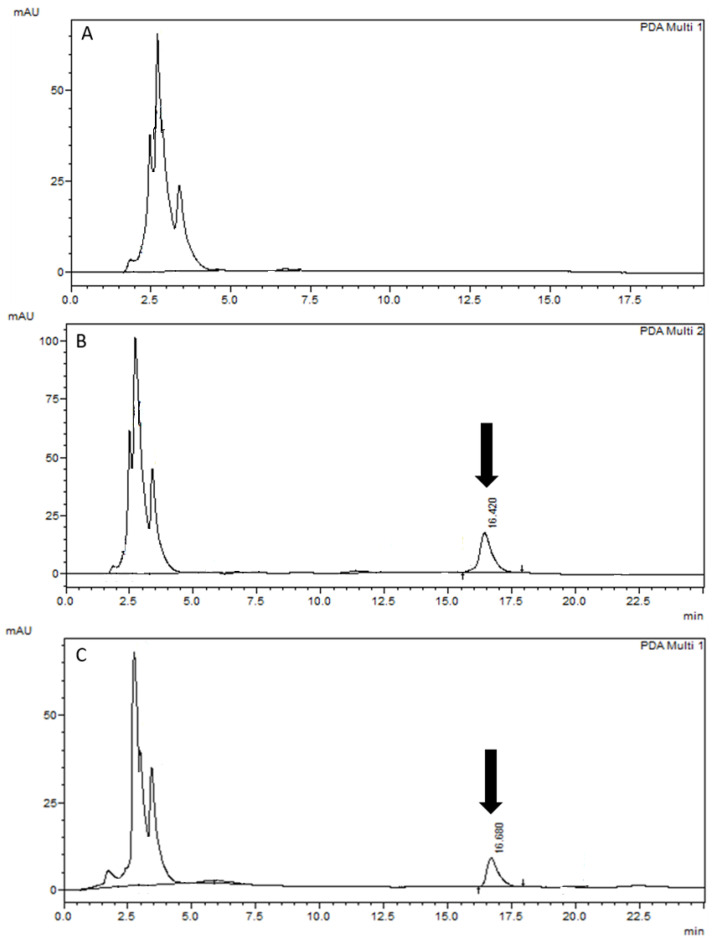
Chromatograms of a rat plasma blank sample observed at λ = 228 nm (**A**), a rat plasma spiked with 4 µg/mL of 6-methoxyseselin (**B**), and a rat plasma obtained at 1 h following intravenous administration of 6-methoxyseselin in rat 15 mg/kg (**C**). The 6-methoxyseselin peak is shown by the arrows.

The selectivity of the method was proven to be adequate. The 6-methoxyseselin peak came out at a retention time (approximately 16.4 min) distant from the endogenous peaks of the white rat plasma (Figure 3A,B). In the rat plasma samples after intravenous administration, there was no interference between the analyte and endogenous peaks (Figure 3C). Thus, the method developed was selective for quantifying 6-methoxyseselin in the plasma samples in pharmacokinetic studies.

### 3.2. Sensitivity

The method’s sensitivity was evaluated by determining the LLOQ of 0.15625 µg/mL, as the intra- and inter-day precision and accuracy were within the acceptable range (CV within ±20% and RSD ≤ 20%; Table 1). The low value of LLOQ indicates that the method is sensitive to quantifying low concentrations of 6-methoxyseselin in preclinical pharmacokinetic studies.

### 3.3. Accuracy and Precision

The accuracy, represented by RSD, and the precision, represented by CV, were evaluated at four intra- and inter-day concentrations (Table 1). Precision testing was performed to assess the repeatability of the method. The mean values of the QC samples (LQC, MQC, and HQC) were within the allowable range (±15%), and for the LLOQ they were within ±20%, indicating that the method was suitable for the quantification of the actual analyte concentrations in the plasma samples.

### 3.4. Linearity

**Figure 4:**
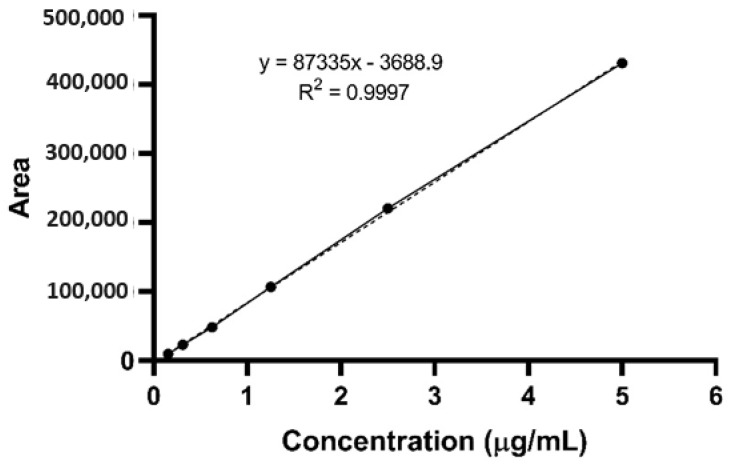
Average calibration curve at a concentration ranging from 0.15625 to 5 µg/mL.

The method was proven to be linear for the quantification, with a typical linear regression equation y = 87335x − 3688.9 (R^2^ = 0.9997), which was higher than 0.99 in the average of the calibration curves (n = 6) in the range of 0.15625–5 µg/mL (Figure 4). In addition, the calibration curve concentrations did not exceed the limit of ±15% of the nominal concentrations and ±20% for the LLOQ (Table 2), indicating adequate linearity for quantifying concentrations expected by the pharmacokinetic study and a good description of the concentration responses.

### 3.5. Recovery

The mean recovery was 112.8% ± 9.2%, 110.1% ± 7.4%, and 122.9% ± 5.6% for LQC (0.5 µg/mL), MQC (1 µg/mL) and HQC (4 µg/mL), respectively (mean ± SD, n = 3). The liquid–liquid extraction used proved efficient, consistent, and reproducible, corroborating the excellent sensitivity presented by the method.

### 3.6. Stability

Stability results (Table 3) show that the samples were considered stable in storage until day 30, showing an RSD within 15%. The LQC of the samples stored for 45 days showed an RSD greater than 15%, and the samples stored for 60 days showed considerable changes in the chromatographic profile, and were considered unstable.

### 3.7. Pharmacokinetics Study in Mice

**Figure 5:**
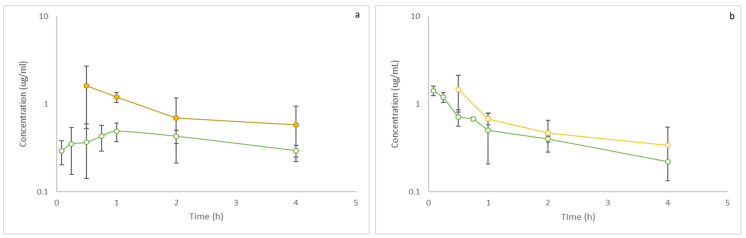
Mean (±SD) 6-methoxyseselin plasma (green) and lung (yellow) concentration vs. time graphs after inhalation (50 mg/kg) (**a**) and IV administration (15 mg/kg) (**b**) in mice (n = 40/total). The *y*-axis is a log scale.

The devised and validated method was proven to be effective in the preclinical pharmacokinetic study. The plasma and lung concentration profiles, depicted in Figure 5, were successfully generated over time. Furthermore, the non-compartmental pharmacokinetic plasma parameters and lung penetration factor were estimated and are presented in Table 4. Notably, the concentration of 6-methoxyseselin remained well within the bounds of the calibration curve and consistently exceeded the LLQQ set at 0.15625 µg/mL.

## 4. Discussion

The selectivity assessment results demonstrate that the method developed to quantify 6-methoxyseselin in plasma samples is suitable for pharmacokinetic studies. The chromatograms exhibited distinct peaks, with the 6-methoxyseselin peak clearly distinguished from the endogenous peaks in both blank and spiked plasma samples, indicating minimal interference between the analyte and endogenous components. This reinforces the method’s accuracy when measuring the 6-methoxyseselin concentrations in rat plasma.

In terms of sensitivity, the method exhibited a low limit of quantification (LLOQ) of 0.15625 µg/mL, enabling precise and accurate detection of low 6-methoxyseselin concentrations, crucial for preclinical pharmacokinetic studies where analyte concentrations can vary widely. Accuracy and precision were evaluated across different concentration levels, yielding consistent results within acceptable ranges. Quality control sample mean values were within ±15% for most concentrations and ±20% for LLOQ, indicating good accuracy and precision. Linearity was confirmed through calibration curves with excellent correlation coefficients and minimal deviation from the nominal concentrations, supporting the method’s reliability for quantifying 6-methoxyseselin in mice plasma samples.

Additionally, the method’s satisfactory recovery rate suggests the efficiency and consistency of the liquid–liquid extraction process. However, stability testing revealed limitations, particularly in long-term storage conditions, underscoring the importance of adhering to proper sample handling and storage protocols in pharmacokinetic studies.

The method was successfully applied to a mice pharmacokinetic study, producing plasma and lung tissue concentration–time profiles. Non-compartmental pharmacokinetic parameters such as area under the curve (AUC), volume of distribution (Vd), clearance (Cl), and half-life (t1/2) were calculated, providing valuable insights into the disposition and elimination of 6-methoxyseselin in vivo. Notably, the lung penetration factor suggests favorable distribution of the compound into lung tissues [19], bolstering its potential as a candidate for treating asthma.

Anti-asthmatic drugs exhibit a spectrum of pharmacokinetic parameters essential for their therapeutic efficacy and safety. These agents demonstrate distinctive pharmacokinetic profiles depending on the routes of administration, protein binding, particle sizes, and type of suspension, among others [20]. Upon absorption, these drugs undergo distribution throughout the organism, with variable volumes of distribution (Vd). For instance, salbutamol typically exhibits a Vd of approximately 3–4 L/kg [21], and budesonide has a Vd of 2.2–3.9 L/kg. The metabolism of anti-asthmatic drugs primarily ensues in the hepatic milieu via diverse enzymatic pathways, yielding variable clearance rates. Renal excretion predominantly mediates the elimination of these agents, with differential half-lives dictating dosing frequencies. Salbutamol has an elimination half-life of approximately 3–4 h [marques], and budesonide has a half-life of 2–3.6 h [22]. These nuanced pharmacokinetic profiles underpin tailored therapeutic strategies, optimizing efficacy while minimizing adverse effects [23]. When comparing the PK parameter values estimated for 6-methoxyseselin, one can infer that they are very similar to the currently used anti-asthmatic drugs, with favorable distribution profiles.

Even though our research aims to assess the efficacy of drug delivery into the lung tissue itself rather than its distribution in the surrounding bronchial tissues or bronchoalveolar lavage fluid, not having the drug’s levels in the bronchial region may limit the overall scope of our findings. One of the reasons for not quantifying the drug levels in the bronchial tissues or bronchoalveolar lavage fluid is the bioanalytical method sensibility.

## 5. Conclusions

This was the first study to develop and validate a 6-methoxyseselin quantification methodology and to evaluate the pharmacokinetics and pulmonary delivery of 6-methoxyseselin in mice. A simple, sensitive, precise, and exact method for determining 6-methoxyseselin in plasma was performed using HPLC-DAD. The methodology allowed for pharmacokinetic study in mice after intravenous and inhalation administration, demonstrating that it is robust and reliable for new preclinical pharmacokinetic studies. Most parameters did not change significantly between the administration routes, but both showed favorable pharmacokinetic profiles. The high penetration factors to the lung after both administrations showed promising results for using 6-methoxyseselin as a new anti-asthmatic drug.

Looking forward, there are several avenues deserving of exploration. Firstly, optimizing the drug formulation, such as utilizing nanoparticles or liposomes, holds promise for enhancing pulmonary delivery efficiency while mitigating systemic exposure. Secondly, conducting comprehensive toxicity studies is imperative to ascertain the safety profile of 6-methoxyseselin. Thirdly, investigating combination therapies involving 6-methoxyseselin alongside existing asthma medications could potentially yield synergistic effects.

## Figures and Tables

**Table 1:** Intra- and inter-day validation results for the determination of 6-methoxyseselin in the mice plasma.

Nominal Concentration Levels	Intra-Day (n = 3) Day 1		Intra-Day (n = 3) Day 2		Inter-Day (n = 6)	
	Precision (CV, %)	Accuracy (RSD, %)	Precision (CV, %)	Accuracy (RSD, %)	Precision (CV, %)	Accuracy (RSD, %)
LLOQ (0.15625 µg/mL)	4.8	−12.6	14.4	3.5	8.4	−4.5
LQC (0.5 µg/mL)	3.1	10.8	6.7	8.9	1.2	9.9
MQC (1 µg/mL)	6.7	10.1	5.6	14.1	2.5	12.1
HQC (4 µg/mL)	7.8	9.2	9.7	3.8	3.7	6.5

Abbreviations: CV, coefficient of variation; HQC, high-quality control; LLOQ, the lower limit of quantification; LQC, low-quality control; MQC, medium quality control; RSD, relative standard error.

**Table 2:** Results of intra-day and inter-day calibration curve in the mice plasma.

Nominal Concentration Levels (µg/mL)	Intra-Day (n = 3) Day 1	Intra-Day (n = 3) Day 2	Inter-Day (n = 6)
	RSD, %	RSD, %	RSD, %
0.15625	−12.6	3.5	−4.5
0.3125	−13.4	4.6	−4.4
0.625	−2.5	−1.7	−2.1
1.25	4.0	−3.8	0.1
2.5	2.5	2.1	2.4
5	−0.8	−0.3	−0.5

Abbreviations: RSD, relative standard error.

**Table 3:** Stability results for the mice plasma.

Concentration Levels	Long-Term Stability (−18 °C, 30 Days) (RSD %)	Long-Term Stability (−18 °C, 45 Days) (RSD %)
LQC (0.5 µg/mL)	11.0	26.6
HQC (4 µg/mL)	0.2	−2.6

Abbreviations: HQC, high-quality control; LQC, low-quality control; RSD, relative standard error.

**Table 4:** Non-compartmental pharmacokinetic parameters of 6-methoxyseselin after the intravenous and inhalation administration of 15 mg/kg and 50 mg/kg, respectively, to mice.

Plasma PK Parameters	Intravenous (Mean ± SD)	Inhalation (Mean ± SD)
AUC inf (h·mg/mL)	3.82 ± 1.69	3.01 ± 1.45
Vd (L/kg)	19.59 ± 1.57	18.79 ± 2.58
Cl (L/h/kg)	5.32 ± 1.23	6.59 ± 2.34
ke (h^−1^)	0.27 ± 0.11	0.21 ± 0.10
t1/2 (h)	2.6 ± 0.73	3.3 ± 0.86
MRT (h)	3.23 ± 1.57	5.4 ± 2.1
Lung penetration factor	1.45	2.14

Abbreviations: AUC, the area under plasma concentration-time curve; Vd, the volume of distribution; Cl, clearance; ke, elimination rate constant; t1/2, half-time; MRT, mean residence time; lung penetration factor = AUC inf lung/AUC inf plasma.

## Data Availability

Most of the data used during the preparation of the manuscript are included in Section 2 and Section 3.
